# Updated evidence of the association between toxocariasis and epilepsy: Systematic review and meta-analysis

**DOI:** 10.1371/journal.pntd.0006665

**Published:** 2018-07-20

**Authors:** Jaime Luna, Calogero Edoardo Cicero, Guillaume Rateau, Graziella Quattrocchi, Benoit Marin, Elisa Bruno, François Dalmay, Michel Druet-Cabanac, Alessandra Nicoletti, Pierre-Marie Preux

**Affiliations:** 1 INSERM, U1094, Tropical Neuroepidemiology, Limoges, France; 2 Univ. Limoges, UMR_S 1094, Tropical Neuroepidemiology, Institute of Neuroepidemiology and Tropical Neurology, CNRS FR 3503 GEIST, Limoges, France; 3 Department of Medical and Surgical Sciences and Advanced Technologies “G.F. Ingrassia”, Section of Neurosciences, University of Catania, Catania, Italy; 4 Sobell Department of Motor Neuroscience and Movement Disorders, Institute of Neurology, UCL, London, United Kingdom; 5 CHU Limoges, Centre d’Epidémiologie de Biostatistique et de Méthodologie de la Recherche, Limoges, France; Instituto de Investigaciones Biomedicas, UNAM /Instituto de Neurologia y Neurocirugía, MEXICO

## Abstract

**Objective:**

To gain further insight on the association between human toxocariasis and epilepsy in light of the new evidence in the last years.

**Methods:**

A systematic review was conducted without date and language restriction in the following electronic databases: MEDLINE (PubMed), Ingenta Connect, Science Direct (Elsevier), RefDoc, Scopus, HighWire, Scielo and the database of the Institute of Neuroepidemiology and Tropical Neurology of the Limoges University (IENT). Two investigators independently conducted the search up to November 2017. A pooled odds ratio (OR) was estimated using a random effects model. Meta-regression was conducted to investigate potential sources of heterogeneity.

**Results:**

Database search produced 204 publications. Eleven case-control studies were included that were carried out in 13 countries worldwide. A total number of 4740 subjects were considered (2159 people with epilepsy and 2581 people without epilepsy). The overall pooled OR was 1.69 (95% CI 1.42–2.01) for the association between epilepsy and *Toxocara* spp. seropositivity. A positive association was constantly reported in the restricted analysis (WB as confirmatory or diagnostic test, younger population, and population–based studies). Meta-regression showed no statistically significant association between covariates and outcome.

**Conclusion:**

The updated meta-analysis provides epidemiological evidence of a positive association between *Toxocara* seropositivity and epilepsy. New surveys supported the association, mainly population-based studies. On this basis, health strategies to reduce the impact of *Toxocara* spp are strongly advised. Further research should be performed to understand the physiopathological mechanisms of *toxocara*-associated epileptogenesis.

## Author summary

Human toxocariasis is a parasitic zoonosis caused by the larval stages of *Toxocara canis (T*. *canis)*, the common roundworm of dogs, and by the roundworm of cats, *Toxocara cati* (*T*. *cati*). Toxocariasis is one of the most prevalent helminthiasis worldwide. Humans become infected by the ingestion of contaminated food or soil or by direct contact with dogs and cats. Epilepsy is the most common chronic neurological disorder worldwide with a global estimate of about 70 million cases of lifetime epilepsy. Early studies suggested high rate of toxocara exposure (toxocara seropositivity) among people with epilepsy. Despite several studies support the possible role of toxocariasis in the incidence of epilepsy, this association is still debated. We updated our previous research to gain further insight on the association. This study provides new evidence of a positive association between the two diseases. On this basis, health strategies to reduce the impact of *Toxocara* spp are strongly advised. Further studies are necessary to clarify the role of certain parasites in epilepsy.

## Introduction

Toxocariasis is a parasitic zoonosis caused by larvae of *Toxocara canis* and *Toxocara cati*, the common roundworms of dogs and cats, respectively.

Toxocariasis is one of the most prevalent helminthiasis worldwide. Even if the parasite tends to be more prevalent in tropical settings where seroprevalence reaches up to 80–90%, in Western countries seroprevalence ranges from 35 to 42% in rural areas and from 2 to 5% in urban areas [1].

*Toxocara* is a nematode that usually inhabits the small intestine of the host. The female *Toxocara* produces up to 200,000 eggs per day, releasing them to the environment through the feces [2]. Humans become infected by direct contact with dogs or by the ingestion of contaminated food or soil. Ingested eggs develop into juvenile larvae that cross the small intestine and migrate to any organ through the circulatory system, resulting in a multisystemic inflammatory tissue reaction [3]. *Toxocara* larvae migrates to the liver via the portal circulation, then lungs and left heart, from where they disseminate via the systemic circulation, especially to muscles, optic nerves and, in rare cases, the central nervous system [4]. Migrating larvae are attacked by host immune responses, resulting in local inflammation associated with eosinophilia and increased production of cytokines and specific antibodies [5].

Soil is considered as one of the main source of transmission to human beings. The reported prevalence of soil contamination with *Toxocara* spp. eggs is variable between studies, going from a percentage of 6.6 to 87.1% [6]. Infection could also occur as accidental ingestion of embryonated eggs from contaminated water, vegetables, fruit [7], raw or undercooked meat or organs from paratenic hosts (cows, sheeps or chickens) [8,9]. Additionally, recent studies found a percentage of dogs and cats contaminated with *Toxocara spp*. eggs in their hair that could be a potential risk factor for the transmission of this parasite to other animals and humans [10,11].

The clinical spectrum of toxocariasis in humans varies from asymptomatic infection to severe organ injury [12]. However, the vast majority of infections remain undiagnosed due to the asymptomatic, mild or non-specific clinical nature of infections [13]. Two severe conditions, visceral larva migrans (VLM) and ocular larva migrans (OLM), and two less severe, “covert toxocariasis” and “common toxocariasis” have been described in the literature [14]. *Toxocara* larvae can cross the blood–brain barrier, invading the central nervous system (CNS), leading to neurotoxocariasis [4,15].

Since the first reported case of nematode larvae migration to the brain by Beautyman and Woolf in 1951 [16], more than one hundred cases of neurotoxocariasis have been reported up to date [4,15]. CNS infestation of *T*. *canis* larvae in humans is thought to be rare, even if in animal models larvae often migrate to the brain [17]. Clinical involvement of the CNS consists of a wide spectrum of neurological manifestations ranging from meningitis, encephalitis, and myelitis, to cerebral vasculitis [4,15].

Early epidemiological reports suggested high seropositivity rates for *T*. *canis* among people with epilepsy (PWE) [18,19]. Following these preliminary observations several case–controls studies have been carried out suggesting a possible role of toxocariasis in the incidence of epilepsy [6].

An accurate estimate of the association between toxocariasis and epilepsy is needed considering that toxocariasis is one of the most common helminthiasis infections worldwide and it is a potentially preventable disease [7]. For this reason, in 2012 our research teams performed a systematic literature review and a meta-analysis of all the available data [6], finding a positive association between *Toxocara spp*. seropositivity and epilepsy. Despite several studies support the possible role of toxocariasis in the incidence of epilepsy, this association is still debated [1]. Considering that new studies have been performed, we updated our previous research to gain further insight on the association between human toxocariasis and epilepsy and to evaluate any methodological improvement in the field.

## Methods

### Search strategy

We updated our previous literature search [6]. The aim was to identify any new published and unpublished evidence on the association between toxocariasis and epilepsy. The new systematic search has been independently conducted by two investigators (JL and CEC) in the following electronic databases: MEDLINE (PubMed), Ingenta Connect, Science Direct (Elsevier), RefDoc, Scopus, HighWire, Scielo and the database of the Institute of Neuroepidemiology and Tropical Neurology of the Limoges University (IENT).

To search for the association between epilepsy and toxocariasis the following research strings and Boolean operators have been entered in each database: “epilepsy” AND “toxocariasis” AND “epidemiology”. In order to find all the available articles, the search term “toxocarosis” has also been used. The research has been performed without date or language restriction. Titles and abstract have been screened to select relevant studies, which have been fully read. For all the selected articles references have been searched for other relevant articles. When necessary, corresponding authors were contacted. Experts in the field were also contacted to find out other eventual non-published studies. The search was realized up to November 2017.

### Study selection

Considering epilepsy as the outcome and toxocariasis as the exposure, studies had to fulfill the following inclusion criteria, already used in the previous meta-analysis [6], to be included:

1. Presence of a control group (people without epilepsy, PWOE);
2. Information about methods used to assess epilepsy;
3. Serological or histopathological detection of toxocariasis;
4. Information about methods and criteria used for case finding and control selection;
5. Possibility to determine the sample size of each of the following four groups in aggregated data: people with epilepsy seropositive for toxocariasis (PWE *Toxocara* spp.+), people with epilepsy not seropositive for toxocariasis (PWE *Toxocara* spp.-), people without epilepsy seropositive for toxocariasis (PWOE *Toxocara* spp.+), people without epilepsy not seropositive for toxocariasis (PWOE *Toxocara* spp.-).

Systematic reviews, case reports or commentaries were not included in the study selection. Surveys including only acute symptomatic seizures or specific seizure patterns or epileptic syndromes were excluded.

### Data extraction

Two investigators (JL and CEC) independently examined the identified articles. Discrepancies were discussed and agreement was reached for all the articles included in the analysis. For each survey the following information have been entered in an *ad hoc* created database: author, country, study design, study population (number, age groups, and gender), study setting, recruitment methods, epilepsy definition and confirmation methods, and exposure assessment.

Association between epilepsy and toxocariasis has been calculated for each included survey giving a crude odds ratio (OR) and their relative 95% confidence interval (CI). Furthermore, *a priori* and *a posteriori* statistical power have been calculated. *A priori* statistical powers were calculated following the hypothesis that the objective of the survey was to identify a minimum OR of 2 with one control per case, based on the number of PWE and the percentage of *Toxocara* spp. seropositivity in PWOE. *A posteriori* statistical power were calculated upon the results of the surveys. In both cases a 5% alpha risk was considered. Powers were calculated using Epi-Info statistical software 6.4. [20]

### Meta-analysis

To estimate the association between epilepsy and toxocariasis a meta-analysis has been performed using a random effects model [21]. The Cochran Q test and I^2^ were used to examine statistical heterogeneity in the meta-analysis [22]. Further restricted analyses have been conducted: i) We applied the analysis only to the studies using Western Blot (WB) as diagnostic or confirmatory test for toxocariasis [23–27], considering that WB is as sensitive but more specific than enzyme-linked immunosorbent assay (ELISA) [28]. ii) We performed a restricted analysis of studies including young population (<18 years) [29–31], in order to account for the different age groups considered. iii) We also conducted a restricted analysis of the studies performed in general population settings [23,27,32], taking into consideration that population-based case–control studies can avoid selection biases compared with hospital-based studies [33]. The publication bias was evaluated through the funnel plot and the Egger’s test was used for detecting asymmetry.

Meta-regression was conducted to investigate potential sources of heterogeneity. We used random effects meta-regression to examine whether any covariate was associated with the outcome. Several study level co-variables were considered. For each meta-regression, the p value and the tau^2^ were reported for each covariate.

The meta-analysis and meta-regression were performed using the statistical software Stata v11.1 (Stata Corporation, College Station, TX, USA). The PRISMA (Preferred Reporting Items for Systematic reviews and Meta-Analyses) statement [34] was followed as a guide to report this study (PRISMA checklist—S1 File).

## Results

### Literature search

**Fig 1:**
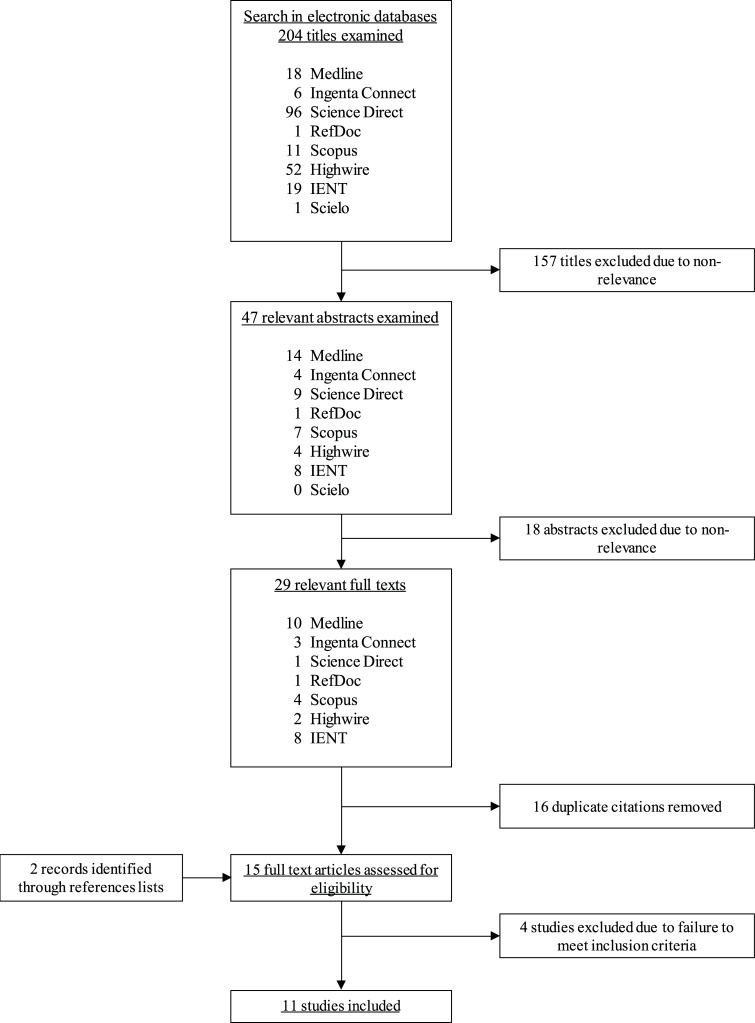
Articles identification and selection flowchart.

The detailed steps of the updated literature research and selection process are shown in the Fig 1. A PRISMA flowchart is also shown (S1 Fig). Database search produced 204 publications. After title and abstract examination 29 articles full text were selected. Searching through references produced additional two publications [31,35]. The removal of duplicates permitted to identify 15 documents. Full text review of the documents allowed us to exclude four of them for not fulfilling the inclusion criteria. In particular, one publication was excluded since there was no control group [19]. Another two publications were also excluded because they provided insufficient information about the methodology used in the surveys [36,37]. We found two new studies from the same research group, and we excluded the first one from the analysis [38] because the same population was considered [32]. Nevertheless, the materials and methods of this last study had been previously detailed in the first publication [38], therefore we assessed the methodological aspects considering both publications. Briefly, 11 studies were considered for the analysis.

### Included studies

Eleven case-control studies were included, seven from our previous systematic review [23–26,29,30,39] and four new ones [27,31,32,35]. A total number of 4740 subjects were considered, 2873 additional subjects compared to our previous meta-analysis (1867 subjects) [6]. This updated study considers more than twice as many cases and controls (2159 PWE and 2581 PWOE) compared to our previous study (850 PWE and 1017 PWOE) [6].

**Fig 2:**
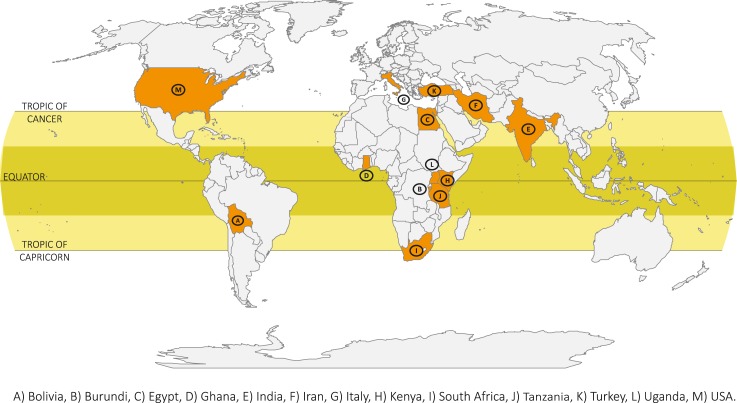
Countries of the studies included on the association between toxocariasis and epilepsy.

The studies were carried out in 13 countries: one in North America [USA] [29], one in South America [Bolivia] [23], two in Europe [Italy] [25,30], three in Asia [India [27], Iran [35], Turkey [39]] and four in Africa [Burundi [24], Egypt [31], Tanzania [26], one of them included 5 African countries: Ghana, Kenya, South Africa, Tanzania, Uganda [32]] (Fig 2). Four studies were performed in rural [23,24,26,32] and seven in urban settings [25,27,29–31,35,39].

The large majority of the surveys were hospital-based studies [24–26,29–31,35,39] and only three population-based case-control studies were found [23,27,32]. Three studies were conducted on a younger population: two considered a population aged 1–17 years [29,30] and one included patients aged 14 years or younger [31]. Seven studies considered no age restriction [23–25,27,32,35,39] while another one excluded children aged 10 years or younger [26]. The general characteristics of the included studies are shown in Table 1.

**Table 1:** Description of the studies included looking for an association between toxocariasis and epilepsy.

			PWE Ascertainment			PWOE		Exposure
References	Country	Study design	Sources	Epilepsy definition and classification	Confirmation	Sources	Matching Criteria	Examinations
Glickman et al., J Pediatr 1979 [29]	USA	Case-control	Pediatric Hospital	Alter et al., 1972	Cases known by hospital	Outpatients or hospitalized controls	None	Sera Ab-ELISA
Arpino et al., Epilepsia 1990 [30]	Italy	Case-control	Pediatric Hospital	Not specified (“positive seizure history”)	Cases known by hospital	Pediatric Hospital	None	Sera Ab-ELISA
Nicoletti et al., Neurology 2002 [23]	Bolivia	Case-control	General population	ILAE 1993; ILAE 1981	Neurologist	General population	Sex, age ± 5 years, same community	Sera Ab- ELISA, WB
Akyol et al., Seizure 2007 [39]	Turkey	Case-control	Hospital, consecutively enrolled	Not specified (“idiopathic epilepsy”); ILAE 1981	Cases known by hospital	Volunteers, source not specified	None	Sera Ab-ELISA
Nicoletti et al., Epilepsia 2007 [24]	Burundi	Case-control	PWE identified by local health centers	ILAE 1993; ILAE 1981	Neurologist	Controls coming to hospital for vaccination or PWE neighbors	Age ± 5 years, no blood relationship, same province	Sera WB
Nicoletti et al., Epilepsia 2008 [25]	Italy	Case-control	Epilepsy center, randomly selected	ILAE 1993; ILAE 1981	Neurologist	Subjects who went to hospital for hematological check, consecutively enrolled	Group-matched by age	Sera WB
Winkler et al., Trans R Soc Trop Med Hyg. 2008 [26]	Tanzania	Case-control	Hospital, age > 10 years	WHO (1993), Winkler et al., 2007	Neurologist	Relatives and volunteers	None	Sera Ab-ELISA, WB, CSF Ab- ELISA
Singh et al., Epilepsia 2012 [27]	India	Case-control	General population	ILAE 1993	Epileptologist, EEG, MRI	General population	Sex, age ± 2 years for age > 10, age ± 1 year for age ≤ 10, same area	Sera Ab-ELISA, WB
El-Tantawy et al., Ajidm 2013 [31]	Egypt	Case-control	Pediatric Hospital	Cryptogenic epilepsy; ILAE Commission on Classification and Terminology, 2005–2009	Cases known by hospital	Volunteers outpatients with no personal or family history of seizures	Age: three age groups (≤5 years, >5 to ≤ 10 years, > 10 to ≤ 14 years)	Sera Ab-ELISA
Zibaei et al., Hindawi 2013 [35]	Iran	Case-control	Hospital	Not specified (“idiopathic epilepsy”) ILAE 1981	Cases known by hospital	Volunteers (healthcare workers or patients’ relatives)	None	Sera Ab-ELISA, WB
Kamuyu et al., PLOS NTD 2014 [32]	South Africa, Tanzania, Uganda, Kenya, Ghana	Case-control	General population	Edwards et al. 2008, ILAE 1993	Neurologist	General population	Group-matched by age, same country	Sera Ab-ELISA

Ab-ELISA, antibodies enzyme-linked immunosorbent assay; CSF, cerebrospinal fluid; ILAE, International League Against Epilepsy; PWE, people with epilepsy; PWOE, people without epilepsy; WB, Western Blot; WHO, World Health Organization.

Four studies [23–25,27] used the epilepsy definition proposed by the International League Against Epilepsy (ILAE) in 1993 [40]. One survey [32] adopted the definition given by *Edwards et al*. in 2008 [41], based on the ILAE active epilepsy definition. Three studies adopted specifics definitions: *Glickman et al*. [29] considered the definition proposed by *Alter* [42] in 1972. *Arpino et al*. [30] considered a general definition of “positive seizure history” as cases entry criteria and *Winkler et al*. [26] defined epilepsy according to the World Health Organization (WHO) Neurosciences Research Protocol proposal [43]. Cryptogenic epilepsy was investigated in three surveys using the ILAE definition [31,35,39].

Considering the type of seizures, five studies [23–25,35,39] applied the classification of epilepsies and epileptic syndromes proposed by the ILAE in 1981 [44], while only one [26] used an adjusted classification for rural African hospitals suggested in 2007 [45]. A standard electroencephalography (EEG) recording was performed in five studies [23–25,27,30] in order to determine the accuracy of seizures classification in patients with a clinical diagnosis of epilepsy.

A neurologist, or an epileptologist, confirmed the epilepsy diagnosis in six studies through anamnesis and complete neurological examination [23–27,32]. All PWE were prevalent cases and only three of the studies clearly specified the inclusion of active epilepsy cases [24,27,32].

Controls have been recruited from the general population in the population-based surveys [23,27,32]. Controls were selected from the same geographical area than cases (rural community or province), and close relatives were excluded by choosing people from different households [23]. In the hospital-based studies, controls were outpatients [30] or in-patients attending the same hospital of cases [29] or people going to hospital for vaccination or blood tests [24,25] or volunteers or relatives [26,31,35,39]. A negative history for seizures [24–27,30,31,35,39] and for both seizures and other neurological diseases [24,25,27,30,35] was the inclusion criteria for controls. Six surveys had a matched case-control design [23–25,27,31,32], age was the most common matching criteria.

Sociodemographic variables and possible risk factors were collected in eight studies [24,25,27,29–32,39] using a questionnaire administered to cases and control subjects.

Several technical approaches were used to assess the presence of anti-*Toxocara* spp. antibodies in sera as antibodies-ELISA (Ab-ELISA) commercial or in-house kits [29–32,39], or Western Blot (WB) [24,25] or Ab-ELISA screening followed by WB confirmation [23,26,27,35]. Laboratories performing the analysis were blind to the case-control status of sera samples in three studies [23–25].

### Association between toxocariasis and epilepsy

Statistical significant association between *Toxocara* spp. seropositivity and epilepsy was found in five surveys [23,25,29,30,32]. Significant crude ORs ranged from 1.58 to 2.85. Borderline statistical significance was found in two studies [24,35]. *A posteriori* statistical power was higher than 80% in only three studies [23,25,32]. *A priori* and *a posteriori* statistical power and crude ORs of the included studies are shown in Table 2.

**Table 2:** Results of the included case-control studies on the association between toxocariasis and epilepsy.

References	PWE (n)	PWOE (n)	Seropositivity in PWE (n)	Seropositivity in PWOE (n)	Seropositivity in PWE (%)	Seropositivity in PWOE (%)	A priori statistical power (%)*	A posteriori statistical power (%)	OR (95% CI)	p-value
Glickman et al., J Pediatr 1979 [29]	84	108	19	11	22.6	10.2	33.6	65.3	2.58 (1.15–5.77)	0.018
Arpino et al., Epilepsia 1990 [30]	91	214	20	26	21.9	12.1	40.1	59.3	2.04 (1.07–3.88)	0.028
Nicoletti et al., Neurology 2002 [23]	113	233	28	28	24.8	12.0	47.6	85.0	2.41 (1.35–4.31)	0.002
Akyol et al., Seizure 2007 [39]	100	50	12	4	12.0	8.0	32.8	11.3	1.57 (0.48–5.14)	0.454
Nicoletti et al., Epilepsia 2007 [24]	191	191	114	97	59.7	50.8	90.9	41.0	1.43 (0.96–2.15)	0.080
Nicoletti et al., Epilepsia 2008 [25]	231	201	38	13	16.4	6.5	55.8	89.6	2.85 (1.47–5.51)	0.001
Winkler et al., Trans R Soc Trop Med Hyg. 2008 [26]	40	20	19	8	47.5	40.0	33.5	8.0	1.36 (0.46–4.03)	0.582
Singh et al., Epilepsia 2012 [27]	106	106	5	6	4.7	5.7	27.0	4.9	0.83 (0.24–2.79)	0.757
El-Tantawy et al., Ajidm 2013 [31]	132	60	64	28	48.5	46.7	79.2	4.3	1.08 (0.58–1.98)	0.815
Zibaei et al., Hindawi 2013 [35]	85	85	10	3	11.8	3.5	16.0	52.0	3.64 (0.97–13.75)	0.043
Kamuyu et al., PLOSntd 2014 [32]	986	1313	308	293	31.2	22.3	100.0	99.8	1.58 (1.31–1.91)	<0.001

*Statistical power assuming an odds ratio equal to 2 with a type I error equal to 5% and one control per case.

OR, crude odds ratio; PWE, people with epilepsy as cases; PWOE, people without epilepsy as controls; seropositivity, presence of antibodies anti-Toxocara canis; 95% CI, 95% confidence interval.

### Meta-analysis

**Fig 3:**
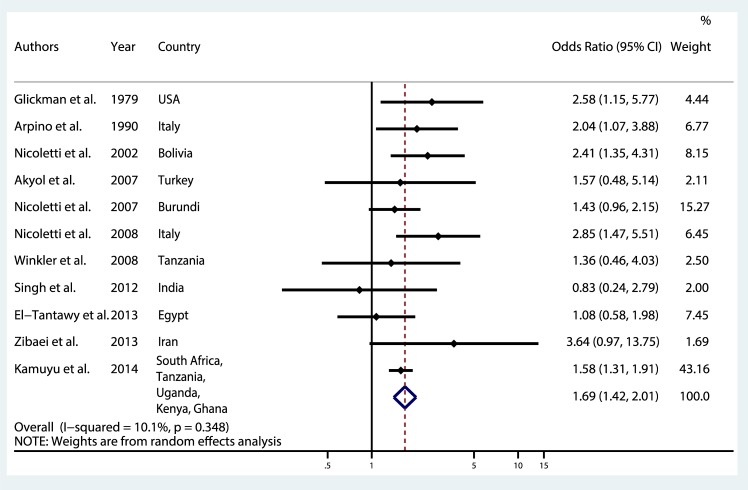
Meta-analysis of studies on the association between toxocariasis and epilepsy.

A meta-analysis was performed considering the 11 studies included. The overall pooled OR was 1.69 (95% CI 1.42–2.01) for the association between epilepsy and *Toxocara* spp. seropositivity (Fig 3). The test of heterogeneity was not significant (p = 0.348) and the I^2^ was 10.1%.

**Fig 4:**
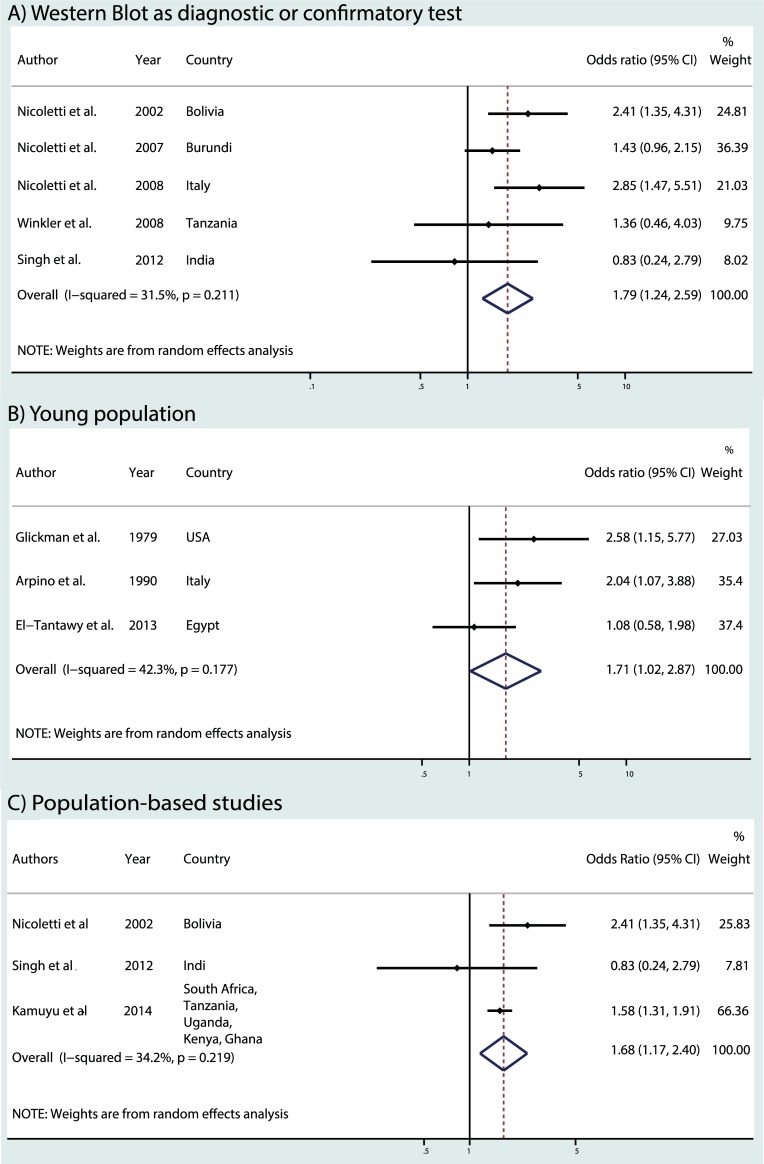
Meta-analysis of studies on the association between toxocariasis and epilepsy: a) Western Blot as diagnostic or confirmatory test, b) young population, c) population-based studies.

Five studies used Western Blot as diagnostic or confirmatory test [23–27,35], we excluded one of them in the restricted analysis because no controls were seropositive using WB [35]. A common OR of 1.79 (95% CI 1.24–2.59) was found [23–27] (Fig 4A). When we restricted the meta-analysis to the three studies considering a young population [29–31], we found a common OR of 1.71 (95% CI 1.02–2.87) (Fig 4B). The meta-analysis was at last restricted to the three population-based studies [23,27,32] leading to an OR of 1.68 (95% CI 1.17–2.40) (Fig 4C). The heterogeneity tests were not significant in the three restricted analyses.

To assess the publication bias, the funnel plot is shown in the S2 Fig and non-significant Egger's test was found (p = 0.454).

### Meta-regression

In order to explore the sources of heterogeneity, the following study level co-variables were considered: time period (tau^2^ = 0.00, p = 0.132), continent (tau^2^ = 0.00, p = 0.181), technique to assess *Toxocara* exposure (tau^2^ = 0.015 p = 0.718), age group population (tau^2^ = 0.015, p = 0.937), case and control ascertainment source (tau^2^ = 0.029, p = 0.813), epilepsy diagnosis confirmation by a neurologist (tau^2^ = 0.011, p = 0.818) and epilepsy definition used (tau^2^ = 0.019, p = 0.809). Meta-regression showed no statistically significant association between covariates and outcome.

## Discussion

Today, the association between toxocariasis and epilepsy is still debated which is related to different results found in the literature and the lack of understanding of physiopathological mechanisms between toxocariasis and epileptogenesis. This updated meta-analysis provides epidemiological evidence of the association between *Toxocara* spp seropositivity and epilepsy, with a pooled overall OR of 1.69 (95% CI 1.42–2.01). The association is consistent but slightly lower than the previous published meta-analysis (OR 1.92, 95% CI 1.50–2.44) [6]. A positive association was constantly reported in the restricted analysis (WB as confirmatory and diagnosis, younger population, and population–based studies).

### New evidence

We updated our previous meta-analysis [6] to gain further insight in the association in light of the new evidence in the last years. We applied similar criteria for the selection of relevant papers in order to include studies with an adequate methodological approach to assess the association.

The four new articles included explicitly investigated the association between epilepsy and toxocara exposition using a case-control design. Case recruitment was performed in a general population setting in two studies [27,32], adopting a two-stage and three-stage design for the detection of cases. For the other two studies, cases ascertainment was performed in a hospital-based setting [31,35]. Controls were selected among the general population of the same communities where the population-based studies were set. In the hospital-based studies, controls have been recruited from the outpatient services of the same hospitals where the cases have been recruited or even from patients’ relatives without epilepsy [35]. A major methodological difficulty with case–control studies is the selection of appropriate control individuals [46]. Selection of population controls ensures that the distribution of exposures in the controls can be readily extrapolated to the population [47]. This is a main point when investigating an association with public health implications. Furthermore, the two population-based studies [27,32] estimated the sample size calculation with satisfactory statistical power (80%).

The new evidence allowed us: i) to consider more than twice as many cases and controls compared to our previous study improving the statistical power to estimate more accurately the pooled OR of the association, ii) to include eight additional countries worldwide which strengthens the consistency criterion for causality, iii) to conduct restricted analysis considering population-based case-control studies.

### Population-based versus hospital-based studies

Overall, the majority of the studies included were performed in a hospital setting. Hospital based studies are frequently prone to selection bias, as the cases being followed in the hospital probably present more severe form of epilepsy compared to the general population. Children suffering from severe encephalopathies, or patients with a history of tonico-clonic generalized seizures more frequently need specialized care in a hospital setting, while subjects presenting milder symptomatology or focal seizures without secondary generalization, might be underrepresented. The difference is even more striking in low and middle-income countries, where the elevated costs of access to specialized care further reduce the representation of milder forms. This bias may partially account for the lack of association found in some of the hospital-based studies, because *Toxocara* spp seropositivity has been showed to be associated with focal seizures (adjusted OR 4.69; 95% CI 2.24–9.80) [25]. Furthermore, controls selected in a hospital-based setting tend to share similar characteristics of the cases, while controls taken from the community better represent the general population. The inclusion of volunteers as controls could be another source of bias since volunteers are frequently healthier than the general population. In order to take into account these sources of bias, we have restricted our analysis to the 3 population-based studies [23,27,32], finding a common OR of 1.68 (95% CI 1.17–2.40), a result close to the one obtained when considering all the studies thus minimizing the impact of the aforementioned selection bias. One of the new population-based studies was a large survey performed in five African nations with an unprecedented sample size and very good *a-priori* and *a posteriori* statistical power [32]. Toxocariasis seropositivity was assessed using Ab-ELISA in sera and it was reported a variable proportion of cases and controls exposure to multiple infections. However, the relative excess risk due to interaction was estimated and adjusted to consider the effect of multiple parasitic infections.

### Exposure to *Toxocara* in young populations

To take in account the possible effect of age on the exposure to *Toxocara* spp, the analysis has been restricted to the studies investigating a young population (<18 years), giving a common OR of 1.71 (95% CI 1.02–2.87), a result now borderline significant when compared to the previous meta-analysis of 2.23 (95% CI 1.36-3-69) [6]. The updated analysis on young population included only one new study published in the last six years, conducted in Egypt by El Tantawy (2013) which found no difference in *Toxocara* spp antibodies rate between cases and controls [31]. Nevertheless, there are some methodological issues to consider as the higher prevalence of subjects with a rural background in the controls compared to the cases. This could raise the number of *Toxocara* spp positive subjects in the control group as living in rural setting increases the possibility of being exposed to *T*. *canis* eggs, especially in a country with a high soil contamination rate such as Egypt [48]. Moreover, the calculated *a posteriori* statistical power was 4.3%, which increases the probability of making a type II error. Age has also been used as matching criteria in all the included studies because it seems to contribute to *Toxocara* spp. exposure. Generally the chance of being exposed to an infectious agent increases with age, however it has also been demonstrated that younger age is associated with higher *Toxocara* spp seropositivity [7] probably due to habits that increase the contact with *Toxocara* spp eggs infected soil (such as playgrounds). Interestingly, toxocariasis is more common in childhood, while neurotoxocariasis cases are observed more frequently in adults [4].

### Epilepsy and types of seizures

Another factor that affects the interpretation of the association between epilepsy and toxocariasis depends on the lack of a clear classification of the etiologies of epilepsies and the different types of seizures. It should be expected, according to the hypothesized physiopathology, that *Toxocara* spp should be associated with focal seizures, with or without secondary generalization rather than generalized seizures. However only few studies provided a stratified analysis according the type of seizures. This association has been confirmed in two of the studies included [23,25], while *Zibaei et al*. (2013) found a borderline significant association in a population comprised largely by patients with partial seizures (82.4%) [35]. Furthermore, seizure have been classified just on a clinical basis in the majority of studies, and accuracy of seizures classification when performed just on clinical ground is questionable. Electroencephalography (EEG) can help differentiating the type of seizures but it is rarely available in epidemiological studies, above all in low and middle-income countries.

### Techniques to evaluate toxocariasis seropositivity

Another possible limitation is due to the heterogeneity of techniques (ELISA, WB, or both) and kits (commercial or in house) used in the different studies to evaluate toxocariasis seropositivity. The standard serological test for the diagnosis of toxocariasis is an ELISA based on secretory-excretory antigens (TES) from *Toxocara canis* larvae of the second stage [49] and it is the most commonly used in epidemiological surveys. ELISA for the detection of specific IgG antibodies to TES in serum has a sensitivity of 78% and a specificity of 92% for the diagnosis of VLM, although cross-reactions with other nematode infections (e.g. *Ascaris lumbricoides*, *Stronglyloides*), reduce its specificity, particularly in tropical areas [50]. The use of fractionated native TES in the western blot (WB) assay overcomes the issues with cross-reactions to other nematodes in ELISA assays [28] thus screening with the indirect TES–IgG–ELISA, followed by confirmation with the TES–WB, is considered an effective approach [5]. Therefore, we have chosen to perform a restricted analysis of those studies that employed Western Blot as confirmatory test. We found a common OR of 1.79 (95% CI 1.24–2.59) that is close to the common OR obtained when considering all the studies.

### Statistical power of the studies

A possible explanation for the lack of association on some of the included studies may rely upon the low statistical power that these studies possessed to find a significant association between epilepsy and toxocariasis. In fact, only three studies had *a posteriori* statistical power over 80%, a value usually considered as appropriate when planning research studies, and they all found the association to be significant [23,25,32]. Nevertheless, studies that had a statistical power between 50% and 80% also found a significant association [29,30,35], while the other studies, which statistical powers ranged from 4.3% to 41.0% did not find any association [24,26,27,31,39].

### Causality

A major limitation in the interpretation of the association is due to the fact that all the studies included in our meta-analysis have been conducted on prevalent epilepsy cases, thus considering a cross-sectional approach at the same time of both the exposure (anti-*Toxocara* spp antibodies) and the outcome (epilepsy). This does not allow us to exclude a reverse causality in which the development of epilepsy acts as a risk factor for the infection with *Toxocara* spp. In fact, epilepsy itself is a risk factor for frequent falls to the ground that increase the chance of *Toxocara* spp infection especially in areas with high levels of soil contamination. Children with epilepsy and mental retardation, such as those suffering from severe perinatal encephalopathies, may have abnormal behaviors (e.g. pica) that increase the chance of *Toxocara* spp infection [29]. To clarify the temporal relation, it is mandatory to investigate the presence of anti-*Toxocara* spp antibodies in a population of incident patients with epilepsy, however no case control study considering incident PWE has been performed to assess the association. A prospective population-based survey including new cases will provide the most reliable epidemiological evidence of association but this does not necessarily imply causation [14]. Therefore, it has been proposed by *Wagner and Newton* (2009) that causal relationship can be established only by measuring the reduction in the incidence of epilepsy following the eradication of the putative helminth (*Toxocara spp*) [51].

### Pathogenic mechanisms: Toxocariasis and epilepsy

The role of exposition to *Toxocara spp* and the development of epilepsy is still a matter of debate. While it has been demonstrated that the parasite can migrate to the CNS and cause a variety of clinical manifestation [14], there are no sufficient evidence supporting a direct epileptogenic role. Some pathogenic mechanisms have been proposed to explain the possible implication of Toxocariasis in epileptogenisis. First, studies have shown that *Toxocara* larvae could induce a granulamotous reaction. An acute granulomatous reaction may cause acute symptomatic seizures, and may leave fibrous scars after resolution and chronic granulomatous lesions maybe could lead to epilepsy [51]. Granulomas has been found in the cortical or sub-cortical brain areas through magnetic resonance imaging (MRI) in neurotoxocariasis cases [15]. Second, animal models have showed that the presence of *Toxocara* spp larvae in the brain is responsible for an increase in the permeability of the blood brain barrier, and the production of proinflammatory cytokines leading to neuronal damage [1]. Similar physiopathological mechanisms have been hypothesized to play a major role in the epileptogenesis due to neurocysticercosis [52] suggesting that the two parasites may share a common epileptogenetic pathway. Third, there is growing evidence that autoantibodies against neuronal elements may play a role in some type of epilepsies [53,54]. Clues of the autoimmune nature of epilepsy came from the presence of antibodies to a major excitatory neuro-transmitter in the CNS [55,56]. Toxocariasis produces a marked immune response including autoantibodies production [57], however it is unclear the relation of these immune mechanisms in epileptogenesis. Further research is needed to clarify the potential implication of these pathways in epilepsy.

### Strengths and limitations

This work relies on certain strengths. First, the broad literature search in eight large databases without date and language restriction, which allow us to screen the available literature in the subject. Second, international guidelines on meta-analysis of observational studies were followed. Third, this update included studies carried out in rural and urban settings in different geographic areas worldwide. Lastly, no apparent publication bias exists in the studies included in the meta-analysis. However, we recognize that the funnel plots should be interpreted with caution when the studies are few.

Some limitations could be discussed. First, only few new studies were performed to evaluate the association, however, this update considers more than twice as many cases and controls compared to our previous study [6]. Second, the statistical power in most of the surveys was well below the minimum of 80% that is considered appropriate. Third, the use of definitions, methodologies and techniques were heterogeneous among the studies. Fourth, certain potential risk factors (pica or exposure to pets) were not systematically assessed. Fifth, relevant clinical features were not always available as epilepsy age at onset and type of seizure. Lastly, we cannot exclude reverse causality because there are no prospective population-based survey including new cases in the literature.

### Public health implications

Human toxocariasis is a cosmopolitan and preventable infection that may have a role in the global burden of epilepsy. There are however many available strategies to reduce the impact of this neglected diseases: public health policies could limit soil contamination and control *Toxocara* infection in definitive hosts [58] and enhancing education will improve the public’s understanding of toxocariasis and its prevention [5]. As an alternative, interest has grown recently to develop vaccines against helminth infections, specifically to prevent transmission of zoonotic diseases to human beings [59].

### Conclusion

We provide epidemiological evidence of a positive association between epilepsy and toxocariasis seroprevalence. New surveys supported the association, mainly population-based studies. We showed that research interest is slightly increasing in the field and studies with appropriate methodological approach have been produced in the last years. We also highlighted the presence of several knowledge gaps that have not been addressed. We strongly encourage researchers to perform further studies in the field to answer these questions. Further research should be performed; ideally, including new cases in a general population settings, to better explore the association or assessing the reduction in the incidence of epilepsy following the eradication of Toxocariasis. Increasing the understanding of physiopathological mechanisms of epileptogenesis will clarify the role of certain parasites in epilepsy.

## Supporting information

S1 File PRISMA checklist. (DOC)

S1 Fig PRISMA flowchart. (EPS)

S2 Fig Funnel plot. (EPS)
